# The Protective Role of Group Activity Prior to COVID-19 Pandemic Quarantine on the Relation between Loneliness and Quality of Life during COVID-19 Pandemic

**DOI:** 10.3390/ijerph20196897

**Published:** 2023-10-08

**Authors:** Adi Vitman Schorr, Itamar Yehuda, Ron Mor

**Affiliations:** 1 Faculty of Science and Technology, Tel-Hai College, Kiryat Shemona 1220800, Israel; itamary@telhai.ac.il; 2 Shamir Research Institute, University of Haifa, Kazrin 1290000, Israel; 3 The Campus for Innovation in Education and Teaching, Tel-Hai College, Kiryat Shemona 1220800, Israel; morron@telhai.ac.il

**Keywords:** physical activity, quality of life, older adults, loneliness feelings

## Abstract

Background: Studies of aging have suggested that QoL is positively associated with active social contacts and supportive face to face social networks. However, social networks and contacts in later life decrease due to a variety of reasons; the narrowing of social networks contributes to increased social isolation and loneliness and leads to the deterioration of well-being and QoL among adult persons. The goal of this study was to explore the relationship between loneliness feelings and QoL during the COVID-19 quarantine as potentially moderated by group physical activity prior to the COVID-19 pandemic quarantine. Methods: A convenience sample of 99 older adults aged 60 and over was interviewed. Using bootstrapping, we tested the strength and significance of the conditional moderation effect of group physical activity prior to the COVID-19 pandemic quarantine on the relationship between loneliness feelings and QoL during the COVID-19 quarantine. Results: the results demonstrated a direct negative effect between loneliness feelings and QoL during the COVID-19 pandemic and that the relationship was moderated by group physical activity prior to the COVID-19 pandemic quarantine (*p* = 0.000). Conclusion: the findings indicate that policy makers and professionals working with older adults should seek ways to attract older adults to participate in group physical activity and enjoy its long-term social benefits.

## 1. Introduction and Literature Review

### 1.1. Background

Recently, researchers in the field of gerontology have been increasingly recognizing that the quality of life (QoL) of adults aged 60 and older is a complex and multifaceted idea that requires in-depth understanding [1]. Studies of aging have suggested that QoL is positively associated with active social contacts and supportive face-to-face social networks [2,3,4]. However, social networks and contacts in later life decrease due to retirement and death of family members and friends [5]. Social networks and social cohesion are important not just in order to maintain one’s social life before retirement but are also found to be part of the success of health intervention programs [6]. Narrowing of social networks contributes to increased social isolation and loneliness [7,8] and leads to the deterioration of well-being and QoL among adult persons [9]. From the other side, researchers showed that engagement in exercise group activities were positively correlated with QoL [10,11]. Others documented the importance of face-to-face social networks for maintaining QoL and decreasing loneliness in later life [5,12].

Loneliness is defined as the perceived gap between actual and desired social relations [11,12]. Although loneliness can be associated with objective indicators of the social network, it is not synonymous with these indicators but rather represents qualitative aspects of the relationships [13]. A substantial body of research has indicated a high level of loneliness as a major risk factor for many health conditions [14,15,16,17]. These negative effects of loneliness might explain the association between loneliness feelings and QoL.

Physical activity (PA) is vastly known for its mental and physical health benefits [15,16,17,18,19,20]. Despite the wide-ranging benefits of participating in regular PA, global inactivity levels are high and increase with age [21,22,23].

In order to promote physical activity among older adults, community organizations run a variety of socially focused physical activity and social programs for older adults. These organizations offer people the opportunity to enjoy a group environment, which can provide social wellbeing benefits such as social connection, reduced perception of loneliness, and possibly increase social support [24]. Socializing in groups is also important for the cognitive and physical health and wellbeing of older adults [25,26]. It was found that physical activity in a group represents an optimal relationship in which the participants derive more significant results [9]. Also, group exercise encourages perseverance over time [27]. In particular, older adults who participate in either sports or hobby groups have a lower risk of the onset of functional disability and better QoL four years later compared to those who did not participate in any group [27].

One potential mechanism is that group participation may strengthen social identification, leading to increased perceived social support [28]. Social support may buffer stressful situations [29] and/or encourage positive health behaviors, including PA [30]. This study holds significance in its exploration of the meaning of group physical activities as a protective mechanism against the potential correlation between loneliness and a decline in quality of life. Moreover, the research clarifies how group physical activities protect against the negative effects of loneliness on quality of life among older adults. It emphasizes the importance of these activities for social connectedness and well-being, and it expands our understanding by revealing that the benefits of group activities persist even after they are temporarily discontinued. This challenges the idea that group dynamics are short-lived.

### 1.2. The Current Study

The current study was conducted in 2020, during the COVID-19 pandemic, which significantly disrupted group physical activity for older adults. Lockdowns and social distancing measures forced the suspension of many group exercise programs, depriving older adults of vital social interactions. As a result of pandemic-related isolation, loneliness among older adults has increased. Many seniors faced reduced social contact, contributing to feelings of loneliness and social disconnection. The data collection was conducted during a COVID-19 pandemic quarantine; in that period, older adults were forced to quit their group physical activity [31], that period and the forced change proved a unique opportunity to assess the effect of the previous group constructive physical activity on the connection between loneliness feelings and QoL among older adults during a particularly stressful period.

**Figure 1:**
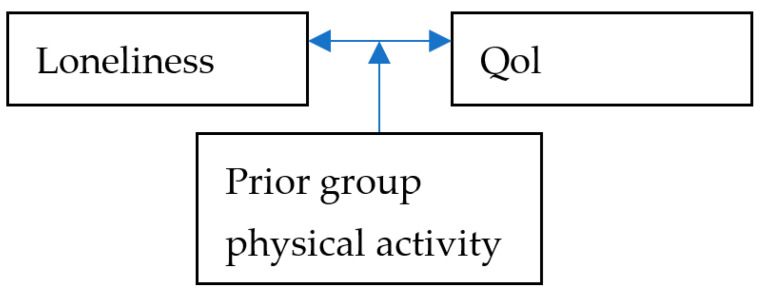
Research model.

We hypothesized that the connection between QoL and loneliness feelings during the COVID-19 quarantine would be moderated by participation in physical group activity prior to the COVID-19 quarantine (Figure 1). Meaning that those who participated in physical group activity prior to the COVID-19 quarantine would not present a connection between loneliness feelings and QoL during the COVID-19 quarantine, while those who did not participate would present that connection.

## 2. Methods

### 2.1. Study Design and Participants

This research employed a cross-sectional study of a convenience sample of 99 older adults in Israel, aged 60 and over; half of the sample used to participate in physical group activity prior to the COVID-19 quarantine, and the other half did not participate. Inclusion criteria were age 60 and over and the ability to speak and understand Hebrew or Russian (by approaching and asking about their degree of familiarity with the languages), independent (determined by the ability to make coherence conversation and ability to answer the questionnaire), and living in the community.

### 2.2. Procedure

This study was approved by the Research Ethics Committee of the college at which the research took place. Recruitment of participants was through convenience sampling, by asking the older adults’ centers for names of participants and non-participants in physical group activity (before the quarantine), the final sample comprised 50 older adults who participated in physical group activity prior (2–3 months before the quarantine) to the COVID-19 quarantine and 49 who did not participate, the sample was composed of Hebrew- and Russian-speaking older adults. Since this research took place when all were quarantined, researchers explained over the phone the study objectives and procedure to the participants, including their right to withdraw freely at any time. Strict confidentiality was maintained. Each participant provided written informed consent by phone message or mail.

Data collection was performed by professional interviewers through telephone interviews, adhering to COVID-19 quarantine restrictions, using appropriate translated, validated, and structured questionnaires. Data collection took place from September to October 2020.

## 3. Measures

### 3.1. Independent Variable

#### Quality of Life

QoL was measured with a 26-item WHOQOL-bref, originating from [32] WHOQOL (1998) using a Likert-type scale 1–5 scoring system. This tool has been widely used [32,33,34]. The total score ranges from 26 to 130, with higher scores indicating better QoL (internal consistency was α = 0.88).

### 3.2. Dependent Variable

#### Loneliness

Loneliness was measured with a 6-item short De Jong Gierveld Scale (2006) [35], using a Likert-type scale 1–5 scoring system. The total score ranges from 6 to 30, with higher scores indicating higher loneliness feelings (internal consistency was α = 0.70).

### 3.3. Moderator

#### Participation in Physical Group Activity Prior to the COVID-19 Quarantine

Participation in group activity was measured with a simple question: “Did you used to participate in physical group activity (low-medium intensity with instructor) prior to the COVID-19 quarantine for at least six months?” Answers were yes (=1) or no (=0).

### 3.4. Covariates

This study controlled for socioeconomic variables. Background variables included gender, age, marital status, self-rated health status, and years of education.

All instruments were translated into Hebrew and Russian by bilingual translators and validated in a pilot study of 10 respondents from each ethnic group (Jews and FSU immigrants). Issues related to both the content and the clarity of the questionnaires were addressed prior to data collection.

## 4. Data Analyses

Descriptive statistics were employed to calculate the means and standard deviations of the continuous variables and the percentage and frequency of the categorical variables. In the second stage, a moderation analysis was performed to examine whether QoL levels during the COVID-19 quarantine were related to loneliness feelings and to determine whether this negative effect was moderated by participation in physical group activity prior to the COVID-19 quarantine (moderation model 1). The moderation analyses were tested using the bootstrap moderation method (model 1) as described by Hayes [36]. With this method, we calculated the conditional effect of independent variables on loneliness at different values (−1 SD, mean, +1 SD) of the potential moderation effects (group physical activity, loneliness feelings, and quality of life) through bootstrapping set at 5000 samples. The analyses were performed using SPSS package version 25.

All analyses were run using SPSS 27.0 with the PROCESS version 4.3 [36]. All estimated effects reported by PROCESS are unstandardized coefficients.

## 5. Results

Of the participants, 32 were women and 67 were men, ranging in age from 63 to 93 (M = 74.8, SD = 7.1). Years of education ranged from 6 to 21 (M = 14.5, SD = 4.4). Self-rated health ranged from 1 to 5 (M = 3.61, SD = 1.06), regarding marital status, 49.5% had a partner. Loneliness feelings ranged between 6 and 30 (M = 14.42, SD = 5.0), quality of life range was 48–115 (M = 90.5, SD = 14.9). Correlation was found between loneliness feelings and participation in physical group activity (Table 1).

## 6. The Moderation Analyses

**Figure 2:**
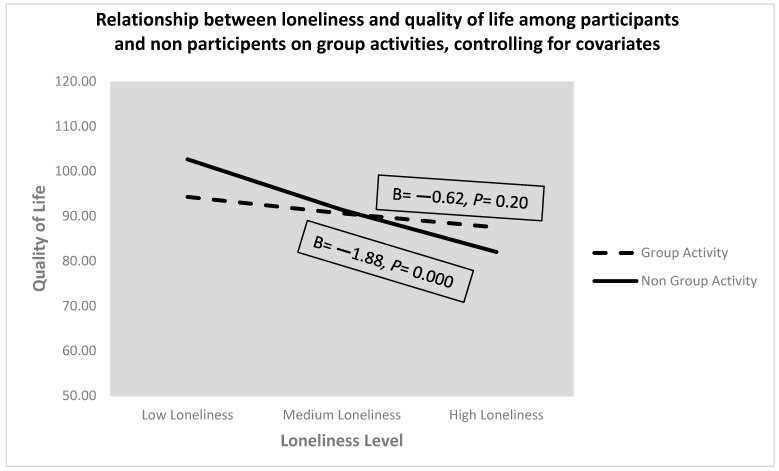
Relationship between feelings of loneliness during the COVID-19 quarantine and quality of life during the COVID-19 quarantine at different levels of physical group activity prior to the COVID-19 quarantine (below average, average, above average), controlling for covariates.

A simple moderation analysis was conducted to explore the moderation effect of participating in physical group activity prior to the COVID-19 quarantine on the connection between feelings of loneliness interaction and QoL during the COVID-19 quarantine. The results show that the slope of the negative relationship between feelings of loneliness during the COVID-19 quarantine and QoL during the COVID-19 quarantine is significant for non-participation in physical group activity prior to the COVID-19 quarantine but not for participating respondents. Evidence for the moderating effect of physical group activity is provided visually in Figure 2.

## 7. Discussion

Physical activity is important for older adults in order to maintain their quality of life through levels of independence, physical and mental health, and well-being [37]. Several studies have shown that physical activity has the potential to maintain older adults’ QOL through the prevention of symptoms of psychological health disorders such as depression and anxiety [38]. Therefore, it is important for older adults to maintain a healthy psychological state through the maintenance of physical activity.

The general purpose of this study was to examine whether participation in physical group activity prior to the COVID-19 quarantine moderated that connection between loneliness feelings and QoL during the COVID-19 quarantine. The findings confirmed the moderation analysis; thus, high feelings of loneliness during the COVID-19 quarantine was related to lower QoL during the COVID-19 quarantine only among those who did not participate in physical group activity prior to the COVID-19 quarantine, while among those who did participate in physical group activity prior to the COVID-19 high quarantine had feelings of loneliness during the COVID-19 quarantine not related to QoL at all. In other words, past physical group activity can protect from very high loneliness feelings (as can be seen in Figure 1) and, as a result, protect from the effect of loneliness feelings on QoL, meaning that those who participated in physical group activity in the past feel less lonely in the present. These feelings of loneliness are not connected to reduced QoL. These results reinforce previous studies that showed the positive associations of physical activity [33] and social contact [39,40] on mental health, feelings of loneliness from one side, and QoL from the other side [18,28,41]. However, the current study shed light on a different phenomenon, since the results show how participating in physical group activity in the near past before the COVID-19 quarantine reduce loneliness feelings in the present and diminish the connection between feelings of loneliness and QoL, even after the physical group activity has stopped and the participants cannot even meet. This finding might be explained by several possible explanations: the first is that group physical activity has a long-lasting effect [27], and the current research shows that this effect is good during crisis times as well; the second possible explanation is that the physical group activity became more than just for physical activity, and some of the participants became personal friends who meet or speak regularly and continued this relationship regardless of the group physical activity, the result of which was canceling the connection between feelings of loneliness and QoL.

## 8. Conclusions and Implications

The primary conclusion is that older adults who used to be engaged in physical group activity prior to the COVID-19 quarantine broke the connection between feelings of loneliness to QoL during the quarantine; the meaning of that is that older adults who engaged in physical group activity in the near past felt less lonely in the present, and as a result, they were protected from reduction in QoL, even when the group activity was stopped. From the perspective of policy and practice, in order to tailor physical activity programs effectively for older adults, it is recommended that surveys be conducted. These surveys should encompass various factors including accessibility, preferred location and timing, as well as the specific activities preferred by this demographic. This approach aims to enhance the accessibility of physical activities and promote homogeneity within groups by aligning with their preferences. Additionally, a complementary measure involves extensive governmental promotion within clinics and adults’ daycare centers, focusing on raising awareness about these activities. Moreover, older adults should know the physical activities’ social benefits for the long run, and not just the short-term benefits regarding the physical and health aspects. Implementation of these measures can be carried out effectively within clinics and care centers. In terms of the scientific implications, this study illuminates the protective role of group physical activity. Further research is warranted to comprehensively grasp and apply this mechanism to older adults, particularly those who may experience prolonged hospitalization or separation from their usual living environment.

## 9. Limitations

We should point out three main limitations of the current study. One is the cross-sectional study design, which does not allow for prediction of a causal relationship between the variables. A further limitation might be the small number of participants. Unfortunately, since the research took place in a very special time, we cannot add participants. Third, a generalization of the findings is limited because the sample and the sampling procedure do not guarantee the representativeness of older adults all around the country. These various factors may have biased the results.

## Figures and Tables

**Table 1:** Descriptive statistics of the study variables (*n* = 99).

Background Characteristics		Total Sample		Participation in Physical Group Activity Prior to the COVID-19 Quarantine			
				Participation *n* = 49	Non-Participation *n* = 50	*p* Value	Effect Size Cohen’s d
**Gender—*n* (%)**	Men		67 (67.7)	9 (49.5)	23 (46.0)	0.003	0.45
	Women		32 (32.3)	40 (50.5)	27 (55.0)		
**Age—Mean (S.D)**		74.8 (7.1)		76.01 (7.1)	73.5 (7.0)	0.22	-
**Education years—Mean (S.D)**		14.5 (4.4)		14.2 (3.6)	14.9 (5.0)	0.05	-
**Self-rated health—Mean (S.D)**		3.61 (1.06)		3.6 (0.94)	3.6 (1.2)	0.17	-
**Marital status—*n* (%)**	No partner		44 (44.4)	20 (40.8)	29 (58.0)	0.08	-
	Has partner		49 (49.5)	26 (53.1)	18 (36.0)		
	Missing value		6 (6.1)	3 (6.1)	3 (6.0)		
**Independent Variable** **Mean (S.D) Range**							
**Loneliness feelings** **range**		14.42 (5.0) 6–30		13.9 (4.3)	14.9 (5.6)	0.04	0.5
**Dependent variable**							
**Quality of life** **Mean (S.D)** **Range**		90.5 (14.9) 48–115		91.6 (12.6)	89.7 (16.5)	0.26	-

## Data Availability

Not applicable.
